# Development and Validation of a Stability-Indicating HPTLC-Based Assay for the Quantification of Nitrofurazone Ointment

**DOI:** 10.3390/molecules30163429

**Published:** 2025-08-20

**Authors:** K. M. Yasif Kayes Sikdar, Hayley Andrews, Kate Pacecca, Aliyah Petker, Sarah Samie, Tomislav Sostaric, Lee Yong Lim, Md Khairul Islam, Cornelia Locher

**Affiliations:** 1 Department of Pharmacy and Centre for Optimisation of Medicines, School of Health and Clinical Sciences, University of Western Australia, Crawley, WA 6009, Australia; yasif.sikdar@research.uwa.edu.au (K.M.Y.K.S.); hayleyandrews2001@gmail.com (H.A.); aliyahpetker001@gmail.com (A.P.); sarahsamie@hotmail.com (S.S.); tom@chromatechscientific.com (T.S.); lee.lim@uwa.edu.au (L.Y.L.); khairul.islam@uwa.edu.au (M.K.I.); 2 Institute for Paediatric Perioperative Excellence, University of Western Australia, Perth, WA 6009, Australia

**Keywords:** HPTLC, ointment, nitrofurazone, validation, stability-indicating assay, quality control

## Abstract

This paper reports on a validated, stability-indicating high-performance thin-layer chromatography (HPTLC)-based assay for the quantification of nitrofurazone in an ointment formulation. The simple and rapid HPTLC analysis was performed on silica gel 60 F254 HPTLC plates using toluene–acetonitrile–ethyl acetate–glacial acetic acid (6:2:2:0.1, *v*/*v*) as the mobile phase and chloroform–acetone (9:1, *v*/*v*) as the solvent. The method was validated in accordance with the guidelines set by both the International Council for Harmonisation (ICH) and the United States Food and Drug Administration (FDA). Nitrofurazone appeared as a sharp band with a R_F_ value of 0.18. The method showed excellent linear regression between the concentration ranges of 30–180 ng/band (*R* = 99.99%). The limit of detection was found to be 10.39 ng/band, and the limit of quantification was 31.49 ng/band. The forced degradation of nitrofurazone via photolysis, oxidation, acid and alkaline hydrolyses confirmed the assay’s suitability for stability studies involving nitrofurazone. Therefore, the method is considered suitable for the routine quality control of nitrofurazone ointment.

## 1. Introduction

**Figure 1:**
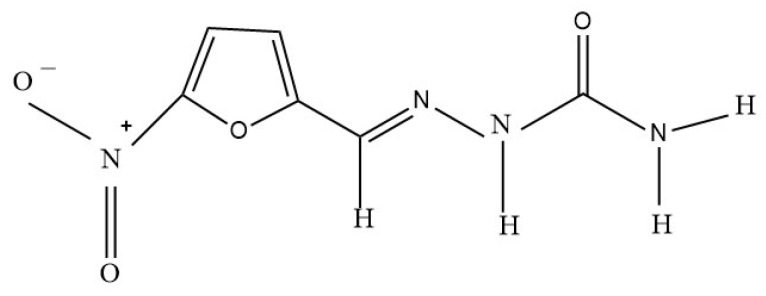
Chemical structure of nitrofurazone.

Nitrofurazone is a broad-spectrum antibacterial agent with a 5-nitrofuran ring and a molecular weight of 198.13 g/mol (Figure 1) [1,2]. It exhibits potent activity against both Gram-negative and Gram-positive bacteria, making it effective for treating burns, wounds, and infections of the skin [2]. However, due to concerns over potential carcinogenic effects, in some countries, nitrofurazone is banned for human use, but is still available in extemporaneously compounded topical ointment formulations for veterinary use [3,4].

Different analytical techniques including UV spectrophotometry, cathodic stripping voltammetry, flow injection chemiluminescence, and spectrofluorimetry have been reported in the literature for the analysis of nitrofurazone in pharmaceutical preparations, whereas high-performance liquid chromatography (HPLC)-UV, liquid chromatography (LC)-diode array detection (DAD), and LC-mass spectrometry (MS) have been used for the detection of nitrofurazone residues in foods and animal feeds [3,5,6,7,8,9,10,11,12]. Moreover, according to the United States Pharmacopeia (USP) monograph, the assay of nitrofurazone can be performed using UV-Vis spectrophotometry, while the assay of nitrofurazone ointment can be carried out using liquid chromatography (LC) with 365 nm detector [13]. Nitrofurazone has also been detected via thin layer chromatography (TLC) and paper chromatography (PC) [4,14]. However, many of the quantitative analytical approaches, including the USP monograph shown in Table 1, require extensive sample pre-treatment and/or extraction procedures prior to analysis, which can complicate the analytical process and necessitate the determination of extraction efficiencies, as well as being time consuming [12,13], whereas TLC and PC are only suitable for qualitative not quantitative analysis [4,14].

High-performance thin-layer chromatography (HPTLC) is a widely utilised technique for the qualitative and quantitative analysis of natural products [15,16,17,18]. It is also increasingly popular in the field of pharmaceutical analysis and quality control as it allows for the parallel analysis of multiple samples, often without the need for pre-analysis extraction, thus enhancing both efficiency and reproducibility [19]. Its simple operation and ability to process numerous samples simultaneously make it a highly cost-effective method for pharmaceutical quality control [19,20]. However, to date, a HPTLC-based assay for the quantitative analysis of nitrofurazone formulations has not been established. This study therefore seeks to develop and validate a stability-indicating HPTLC assay for determining the drug content of a topical nitrofurazone formulation.

## 2. Results and Discussion

### 2.1. Choice of Suitable Solvent System

After a series of trials, which are presented in Section 3.2, chloroform–acetone (9:1, *v*/*v*) was found to be the most effective solvent mixture for dissolving both ointment and nitrofurazone, due to its balanced polarity profile. One gram of ointment (drug content 0.20% *w*/*w*) in 10 mL of optimised solvent yielded a clear solution with no visible particles, indicating that not only the drug but also all ointment components had completely dissolved.

### 2.2. Selection of a Suitable Mobile Phase

Chromatographic test runs with nitrofurazone solution, blank simple ointment, and nitrofurazone ointment demonstrated that each trialled mobile phase effectively separated nitrofurazone from the components of the ointment base, seen as a distinct drug band when the plate was observed at 366 nm (Table 2) (Figure S1). Toluene–acetonitrile–ethyl acetate–glacial acetic acid (6:2:2:0.1, *v*/*v*) achieved the highest degree of separation between the bands and was therefore selected as the mobile phase for the assay.

### 2.3. Method Validation

#### 2.3.1. Specificity

**Figure 2:**
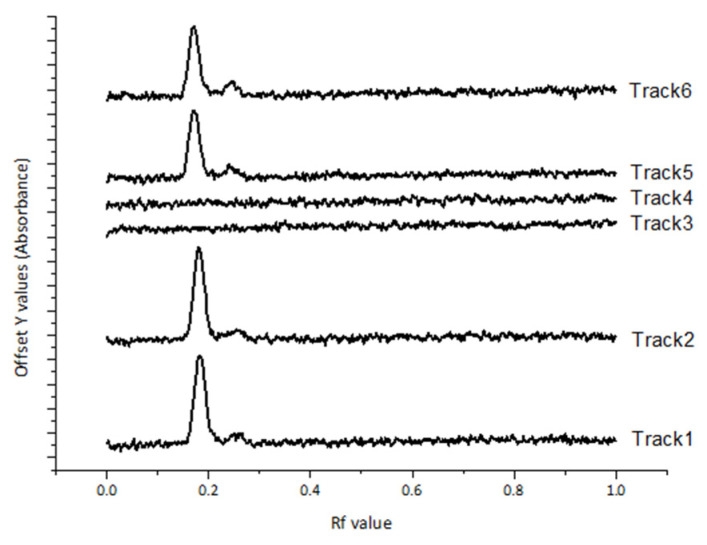
HPTLC chromatogram of nitrofurazone at 366 nm. Track 1 and 2: Nitrofurazone standards; Track 3 and 4: Simple ointment; and Track 5 and 6: nitrofurazone ointment.

The concurrent HPTLC analysis of the nitrofurazone, blank simple ointment, and nitrofurazone ointment solutions demonstrates specificity as nitrofurazone presented as single peak at R_F_ 0.18 with no evidence of any ointment components at 366 nm (Figure 2).

#### 2.3.2. Linearity

A series of nitrofurazone standard solutions were assessed for linearity by deriving a five-point calibration curve. An average determination coefficient of *R*^2^ = 0.9983 indicates a strong linear relationship, which is above the accepted criteria of *R*^2^ = 0.995, as defined by the ICH and FDA guidelines [21,22].

Thus, it can be concluded that the measured response is directly proportional to the concentration of the analyte across the tested range, ensuring the reliability of the method for quantitative analysis. Three separate runs with standards ranging in concentration from 30 to 180 ng/band all demonstrated strong correlation coefficients (Table 3).

#### 2.3.3. Sensitivity

The sensitivity of the method was assessed using trendline linear regression equations from three calibration curves. The LOD and LOQ for nitrofurazone were calculated as 10.39 ng and 31.49 ng per band, respectively (Table 3).

Currently, the most commonly used instrument for the quantification of nitrofurazone in ointments is HPLC. This analytical approach also allows for nitrofurazone detection and quantification with high levels of sensitivity [3]. However, HPLC analysis requires a pre-analysis extraction step, a process that is not required in HPTLC, thus making the latter a more efficient method in terms of simplicity and speed.

#### 2.3.4. Accuracy

The obtained mean sample recovery values of 98.74–100.49% all fall within the range of recovery accepted by the ICH and FDA guidelines [21,22]. The consistent recovery rates across multiple runs and the close alignment of the experimental results with the true concentrations of the analyte in the samples confirm the accuracy of the developed nitrofurazone assay (Table 4).

#### 2.3.5. Precision

Precision was assessed at three levels (80, 100, and 120 ng/band) to reflect the closeness of multiple measurements. For each set of results, shown in Table 5 and Table 6, the %RSD values were within the acceptable range of less than 5% [21,22]. The analytical method can therefore be deemed reproducible with high levels of intra- and inter-day precision.

#### 2.3.6. Repeatability

Repeatability was validated through the analysis of a nitrofurazone ointment (0.20% *w*/*w*) sample solution at six different times. The obtained results (Table 7) were compared with the predicted value, yielding percentage content values with an %RSD of 2.49%, which is below the acceptable value of 5% as defined by the ICH and FDA guidelines [21,22].

#### 2.3.7. Robustness

Deliberate changes to mobile phase parameters were tested to study robustness, the results of which are shown in Table 8. There were no significant changes in the R_F_ value of nitrofurazone, and the % recovery of the drug from the ointment preparation ranged between 95.75 and 104.08% when theoretical concentrations of 80, 100, and 120 ng/band were investigated. This suggests that the method is robust, with little sensitivity towards these types of deliberate changes.

### 2.4. Forced Degradation of Nitrofurazone

**Figure 3:**
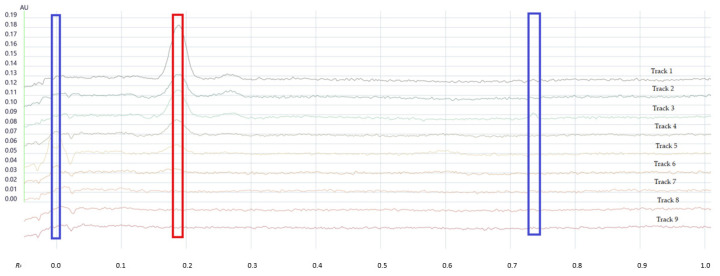
HPTLC chromatogram of forced degradation of nitrofurazone. Track 1: control; Track 2: photolysis and oxidation; Track 3: oxidation; Track 4: acid hydrolysis (0.1 M HCl); Track 5: acid hydrolysis (0.5 M HCl); Track 6: acid hydrolysis (1 M HCl); Track 7: alkaline hydrolysis (0.01 M NaOH); Track 8: alkaline hydrolysis (0.05 M NaOH); and Track 9: alkaline hydrolysis (0.1 M NaOH). Red box—nitrofurazone peak; Blue boxes—degradation products.

Stability-indicating assays are an important part of ongoing pharmaceutical quality control. Considering the inherent instability of nitrofurazone, particularly upon exposure to light and basic pH, a stability-indicating assay is paramount to ensure that the analytical approach can also be used to determine the shelf life of a formulation and monitor its stability over time and under different storage and packaging conditions. Forced degradation studies under photolytic and oxidative conditions, oxidative conditions alone, and in acidic and basic media were therefore carried out to demonstrate accurate stability-indicating quantitative analysis. The HPTLC chromatograms of the forced degradation assays are shown in Figure 3 and the main findings summarised in Table 9.

As can be seen from these results, the area of the nitrofurazone peak decreased in all conditions, including upon UV exposure, which confirms the established photolabile nature of the drug [1]. An acidic environment also degraded the nitrofurazone in a concentration-dependent manner, with 0.1 M, 0.5 M, and 1 M HCl resulting in 19.29%, 24.37%, and 25.38%, respectively. Upon exposure to NaOH at three different concentrations (0.01 M, 0.05 M, and 0.1 M), degradation was strong and rapid, resulting in the absence of any remaining drug peak, which mirrors the findings of basic degradation studies reported by others [3].

The oxidative degradation conditions resulted in a degradation peak (R_F_ 0.735), which was well separated from the drug peak (R_F_ 0.185). A very polar degradation product could also be seen under acidic conditions, evident in a small degradation peak at the baseline. No additional degradants could be detected under any of the investigated conditions across the entire UV-Vis spectrum (200–800 nm), but in all cases, the peak purity of the remaining nitrofurazone peak was confirmed via spectral comparison with the pure drug.

### 2.5. Analysis of Nitrofurazone Ointments

The validated method was used to determine the drug content of an extemporaneously compounded nitrofurazone ointment with a stated drug content of 0.20% (*w*/*w*). The nitrofurazone yield (*n* = 3) was expressed as a percentage of the theoretical concentration (Table 10). The findings revealed that the stated percentage content of the compounded ointment was within the acceptable limits, demonstrating the suitability of the developed HPTLC method for pharmaceutical quality control [21,22]. According to the respective USP monograph, both UV spectrophotometry and liquid chromatography with UV-Vis analysis are used to detect and quantify nitrofurazone in ointment preparations [13]. Compared to the developed HPTLC method, UV-Vis analysis offers only moderate to low sensitivity and specificity levels. Furthermore, HPTLC analysis offers the possibility of running multiple samples on a single plate with minimal solvent input, which might be more cost-effective compared to other chromatographic methods [15]. Thus, the developed HPTLC-based analysis protocol can be considered a suitable alternative analytical approach for the quality control of nitrofurazone ointment formulations, including any stability studies.

## 3. Materials and Methods

### 3.1. Materials and Reagents

All solvents and reagents utilised in the analysis were of analytical grade. Nitrofurazone (>98% purity) was sourced from Tokyo Chemical Industry Co., Ltd. (Tokyo, Japan). Acetone, chloroform, ethyl acetate, and methanol were purchased from Merck KGaA (Darmstadt, Germany), acetonitrile from Sigma–Aldrich (Steinheim, Germany), and glacial acetic acid and toluene from Chem Supply Pty Ltd. (Gillman, Australia). The ingredients for the extemporaneous compounding of the ointment, white soft paraffin BP, wool fat, and hard paraffin, were supplied by PharmAust Manufacturing (Malaga, Australia), and cetostearyl alcohol by PharmAust Manufacturing (Malaga, Australia).

### 3.2. Method Development

Several solvents including ethyl acetate, methanol, cyclohexane, chloroform, cyclohexane–chloroform (1:2, *v*/*v*), cyclohexane–chloroform (2:1, *v*/*v*), cyclohexane–chloroform, (1:1 *v*/*v*), and chloroform–acetone (9:1, *v*/*v*) were investigated for their suitability for adequately dissolving the drug and its ointment base. For this, different quantities (0.30 g, 0.50 g, and 1.00 g) of nitrofurazone ointment and simple ointment (representing the blank ointment base) were dissolved in the respective solvent, sonicated for five minutes to enhance dissolution, and then assessed for the presence of undissolved particles by the naked eye. Chloroform–acetone (9:1, *v*/*v*) was identified as the most suitable solvent for the complete dissolution of both the ointment base and the nitrofurazone load.

The mobile phases tested were formic acid–ethyl acetate–toluene (1:3:1, *v*/*v*), formic acid–ethyl acetate–toluene (1:4:1, *v*/*v*), and toluene–acetonitrile–ethyl acetate–glacial acetic acid (6:2:2:0.1, *v*/*v*). The drug’s resultant retention factors (R_F_ values) as well as the effectiveness of the respective mobile phases to separate the drug band from the ointment component bands were used as criteria to select the most suitable mobile phase. Based on these criteria, toluene–acetonitrile–ethyl acetate–glacial acetic acid (6:2:2:0.1, *v*/*v*) was selected as the mobile phase for the assay.

### 3.3. Compounding of Blank Ointment and Nitrofurazone Ointment

A blank ointment base was compounded using a fusion method by mixing wool fat (5% *w*/*w*), hard paraffin (5% *w*/*w*), cetostearyl alcohol (5% *w*/*w*), and white soft paraffin (85% *w*/*w*) in an evaporating dish and melting over a water bath at 75 °C [23]. The resulting mixture was stirred continuously until homogenous and then allowed to cool to achieve a uniform consistency [23]. Following the fusion method, the nitrofurazone ointment (0.20% *w*/*w*) was prepared by mixing the respective amount of nitrofurazone powder with the blank ointment base. Duplicate nitrofurazone ointments were compounded.

### 3.4. Preparation of Standards and Ointment Solutions

For developing the standard curve, a stock solution was prepared by accurately weighing approximately 15 mg of nitrofurazone in a 250 mL volumetric flask and making up to volume with chloroform–acetone (9:1, *v*/*v*), followed by sonication for five minutes. Five microlitres of this solution was made up to a final volume of 10 mL with chloroform–acetone (9:1, *v*/*v*), resulting in a final stock solution concentration of 30 μg/mL.

To prepare duplicate nitrofurazone ointment sample solutions, approximately 1 g each of the ointment (drug content 0.2% *w*/*w*) was weighed accurately into a beaker. The ointment was dissolved in approximately 10 mL of chloroform–acetone solution (9:1, *v*/*v*). This mixture was transferred to a 50 mL volumetric flask, made up to volume with the solvent, and sonicated for five minutes. Then, 5 mL of this solution was transferred into a 10 mL volumetric flask and made up to volume to obtain a final drug concentration of 20 μg/mL.

The ointment base was prepared using the Simple Ointment APF 25th edition formula. All flasks were covered with aluminium foil to prevent light degradation of the drug [24,25]. New solutions were prepared for each HPTLC run.

### 3.5. HPTLC Method Development

Various volumes (1–6 μL) of the nitrofurazone stock solution were applied to silica gel 60 F254 HPTLC plates (20 cm × 10 cm, Merck, Darmstadt, Germany) as 8 mm bands to construct a standard curve ranging in concentration from 30 to 180 ng/μL. The bands were positioned 8 mm from the lower edge and 11.4 mm from the side edge of the plate. The application was carried out at a rate of 200 nL/s using a semi-automated HPTLC applicator (Linomat 5, CAMAG, Muttenz, Switzerland).

Chromatographic development was carried out in an automated development chamber (ADC2, CAMAG) maintained at 33% relative humidity. Then, 10 mL of the mobile phase (toluene–acetonitrile–ethyl acetate–glacial acetic acid, 6:2:2:0.1, *v*/*v*) was added to the development chamber, and 25 mL was added to the saturation chamber. The chamber was saturated with the mobile phase for 20 min and the plates were conditioned for 5 min before being automatically developed to a distance of 70 mm at room temperature. After a 5 min drying time, the plates were visualised in a CAMAG TLC Visualiser 2 at 254 nm and then scanned at 370 nm, which corresponds to the λ_max_ (TLC Scanner 4, CAMAG) value of nitrofurazone. All processes were controlled using the CAMAG VisionCATS software (v3.1, CAMAG).

### 3.6. Method Validation

The HPTLC assay was validated for specificity, linearity, sensitivity, accuracy, precision, repeatability, and robustness according to guidelines from the International Council for Harmonisation (ICH): Validation of Analytical Procedures Text and Methodology Q2 (R2), and the United States Food and Drug Administration (US FDA), which also recommends adherence to the ICH Q2 (R2) guidelines [21,22].

#### 3.6.1. Specificity

Specificity refers to the method’s capacity to distinctly separate various components within a sample. To evaluate this, tests were performed to determine whether the developed analytical method could accurately detect and quantify nitrofurazone in the ointment formulation. The method’s separation efficiency was confirmed by comparing the R_F_ value of the pure drug with that of the drug in the ointment and by the method’s ability to clearly separate the nitrofurazone band from other ointment constituent bands.

#### 3.6.2. Linearity

The linearity of the procedure was evaluated through linear regression analysis of a five-point calibration curve with a concentration range 30–180 ng/band. Peak areas obtained were plotted against corresponding standard concentrations. The relationship was assessed using the regression equation’s correlation coefficient (*R*^2^), slope (m), y-intercept (c), and the standard deviation (SD).

#### 3.6.3. Sensitivity

The sensitivity of the assay is defined by two key parameters: the limit of detection (LOD) and the limit of quantification (LOQ). The LOD of an individual analytical procedure represents the smallest amount of analyte that can be detected (though not necessarily quantified) under the experimental conditions, whilst the LOQ denotes the minimum amount of analyte that can be reliably detected and quantified with appropriate precision, accuracy, and reproducibility. The calculation of both limits is based on the standard deviation of the response and slope of the calibration curves (*n* = 3), with

LOD = 3.3 × (standard deviation of regression line/slope of calibration curve)

and

LOQ = 10 × (standard deviation of regression line/slope of calibration curve).

#### 3.6.4. Accuracy

Accuracy indicates how closely the results align with the true values. In this work, it was tested through a triplicate recovery study conducted at three different concentrations, namely 80%, 100%, and 120% of the target concentration of 100 ng/band, which was selected because it falls within the calibration curve’s range, ensuring reliable quantification.

#### 3.6.5. Precision

Precision refers to the degree of consistency between the results obtained from multiple analyses of samples under the same conditions. For the current method, precision was validated by analysing three different concentrations in triplicate for both intra-day and inter-day variations. The results are expressed as the percentage relative standard deviation (%RSD).

Intra-day precision assesses the repeatability of the HPTLC analysis carried out three times within the same day under the same conditions. Inter-day precision evaluates the reproducibility of the method between different days to determine if it can consistently produce accurate and reliable results over longer periods despite potential changes in environmental conditions, equipment calibration, or analyst variability.

#### 3.6.6. Repeatability

Repeatability was assessed by analysing a specific sample concentration (80 ng/band) six times. This metric reflects the system’s precision when operating under identical conditions within a short time frame. The results are expressed as %RSD. An 80 ng/band target concentration was chosen as it falls within the calibration curve’s range, allowing for accurate quantification.

#### 3.6.7. Robustness

Robustness refers to the ability of an analytical procedure to maintain consistent performance even when small, deliberate variations are made to method parameters. To evaluate robustness in this study, the effects of altering the composition of the mobile phase on the results were observed. The mobile phase composition was modified from the original toluene–acetonitrile–ethyl acetate–glacial acetic acid ratio of 6:2:2:0.1, *v*/*v* to ratios of 6:1.6:2:0.1, *v*/*v* and 6:2.4:2:0.1, *v*/*v*. For these analyses, 4, 5, and 6 µL of a 20 µg/mL nitrofurazone solution were applied. The amount of drug per band was measured and the % recovery was calculated.

### 3.7. Degradation Studies

A stock solution was prepared by weighing 3 mg of nitrofurazone in a 100 mL volumetric flask and making up to volume with methanol. The stock solution was wrapped in aluminium foil to prevent light degradation.

(i) Control: For the control, 5 mL of this stock solution was made up to a 15 mL volume with deionised water. The solution was covered with aluminium foil and kept at room temperature for 60 min;
(ii) Photolysis and oxidation: A total of 5 mL of 30% H_2_O_2_ was added to 5 mL of the stock solution and the mixture was exposed to ultraviolet (UV) light within a UV chamber for 60 min. The solution was then made up to a volume of 15 mL with deionised water;
(iii) Oxidation: A total of 5 mL of 30% H_2_O_2_ was added to 5 mL of the stock solution and the mixture was covered with aluminium foil and placed in a water bath at 85 °C for 60 min. Once cooled to room temperature, the solution was made up to a volume of 15 mL with deionised water;
(iv) Acid hydrolysis: A total of 1.5 mL of three different concentrations of HCl (0.1 M, 0.5 M, and 1 M) was added to 5 mL of the stock solution separately and the mixture was covered with aluminium foil and placed in a water bath at 85 °C for 60 min. Once cooled to room temperature, 0.1 M of NaOH was added to achieve a pH of 7 (Eutech PC 2700 pH Meter). Each of the solutions was then made up to a volume of 15 mL with deionised water;
(v) Alkaline hydrolysis: Like acid hydrolysis, 1.5 mL of three different concentrations of NaOH (0.01 M, 0.05 M, and 0.1 M) was added to 5 mL of the stock solution separately, and the flasks were then wrapped in aluminium foil and placed in a water bath at 85 °C for 60 min. Once cooled to room temperature, the pH was adjusted to 7 (Eutech PC 2700 pH Meter) using 0.1 M of HCl. Each of the solutions was then made up to a volume of 15 mL with deionised water.

For the detection of any potential degradation products, HPTLC analysis was performed by applying 5 µL of each solution per band, using toluene–acetonitrile–ethyl acetate–glacial acetic acid (6:2:2:0.1, *v*/*v*) as the mobile phase.

## 4. Conclusions

This study introduced a fully validated, stability-indicating HPTLC analysis for the quantitative analysis of nitrofurazone in ointment formulations. The method offers accurate measurement of the nitrofurazone content without the limitation of an extraction step prior to analysis that is necessary in other analytical approaches, including HPLC analysis of these formulations. The developed HPTLC method offers excellent sensitivity, with a LOD and LOQ of 10.39 ng/band and 31.49 ng/band, respectively, and displays high levels of linearity (*R* = 99.99%), specificity, and accuracy, with the findings confirming that the protocol effectively separates nitrofurazone from ointment excipients with consistent results across various concentrations. Moreover, the forced degradation of nitrofurazone means that any potential degradation products do not collapse with the nitrofurazone peak and the band at R_F_ 0.185, confirming the suitability of the method for stability testing of nitrofurazone-containing formulations. HPTLC’s distinct advantages include its operational simplicity and its capability in supporting rapid simultaneous analyses, making the developed HPTLC method a promising stability-indicating assay for the quality control of nitrofurazone ointments.

## Figures and Tables

**Table 1:** Published analytical methods used for analysing nitrofurazone.

Method	Sample Pre-Treatment	Detection	LOD (µg/mL)	LOQ (µg/mL)	Stability-Indicating	Operability	Cost	Type of Sample	References
HPLC-DAD	-	Diode array	0.15	0.49	Yes	Complex	High	Pharmaceutical preparation	[3]
PC	Yes; Water–ethanol (1:2); Centrifugation	UV	-	-	Yes	Easy	Relatively low	Pharmaceutical preparation	[4]
PC	Yes; Water–ethanol (1:2); Centrifugation	UV	-	-	-	Easy	Relatively low	Pharmaceutical preparation	[5]
Spectrophotometer	Yes; DMF and 0.1 M sodium hydroxide solution	Fluorometry	0.0003	0.0010	Yes	Easy	Low	Pharmaceutical preparation	[9]
LC-MS	Yes; Chloroform–acetone (2:1, *v*/*v*)	Mass spectrometer	0.004	-	-	Complex	High	Pasteurised milk	[10]
HPLC-DAD	Yes; Methanol–20 mM sodium acetate (pH 4.6)–acetonitrile (50:40:10, *v*/*v*/*v*) with *n*-hexane	Diode array	2.5	-	-	Complex	High	Avian eggs	[11]
HPLC–MS	Yes; Methanol–20 mM sodium acetate (pH 4.6)–acetonitrile (50:40:10, *v*/*v*/*v*) with *n*-hexane	Mass spectrometer	3.2	-	-	Complex	High	Avian eggs	[11]
HPLC	Yes; Methanol–acetonitrile (1:1, *v*/*v*) containing 1% ammonia solution	UV	2	-	-	Complex	High	Feeds	[12]
LC-MS	Yes; Methanol–acetonitrile (1:1, *v*/*v*) containing 1% ammonia solution	Mass spectrometer	0.2	-	-	Complex	High	Feeds	[12]
LC	Yes; Dimethylformamide and alcohol; Sonication and filtration	UV	-		-	Complex	High	Pharmaceutical preparation	[13]
TLC	-	UV	<1		-	Easy	Low	Standard	[14]

**Table 2:** R_F_ values of nitrofurazone and ointment components in different mobile phases.

Mobile Phase	Nitrofurazone R_F_	Ointment R_F_
Formic acid–ethyl acetate–toluene (1:3:1)	0.51	0.88
Formic acid–ethyl acetate–toluene (1:4:1)	0.67	0.89
Toluene–acetonitrile–ethyl acetate–glacial acetic acid (6:2:2:0.1)	0.18	0.83

**Table 3:** Linear regression data of nitrofurazone standards.

Run	Linearity Range (ng/bands)	Regression Equation	Correlation Coefficient (*R*^2^)	LOD (ng)	LOQ (ng)
1	30–180	y = 3 × 10^−5^x + 0.004	0.9990	10.39	31.49
2	30–180	y = 4 × 10^−5^x + 0.0007	0.9960		
3	30–180	y = 4 × 10^−5^x + 0.0005	0.9998		

**Table 4:** Accuracy of nitrofurazone in ointment.

Theoretical Concentration (ng/bd)	Run 1			Run 2			Run 3		
	Amount Recovered (ng/band)	% Recovery	% Mean Recovery	Amount Recovered (ng/band)	% Recovery	% Mean Recovery	Amount Recovered (ng/band)	% Recovery	% Mean Recovery
80	81.24	101.55	100.43	81.59	101.99	100.49	76.26	95.33	98.74
100	96.07	96.07		98.32	98.32		99.55	99.55	
120	124.40	103.67		121.40	101.17		121.60	101.33	

**Table 5:** Inter-day precision.

Theoretical Amount (ng/band)	Measured Amount (ng/band)			Mean (ng/band)	SD	%RSD
	Run 1	Run 2	Run 3			
80	80.34	81.96	82.30	81.53	0.86	1.05
100	100.50	100.60	100.60	100.57	0.05	0.05
120	124.50	116.10	121.90	120.83	3.51	2.91

**Table 6:** Intra-day precision.

Theoretical Amount (ng/band)	Measured Amount (ng/band)			Mean (ng/band)	SD	%RSD
	Run 1	Run 2	Run 3			
80	74.72	78.35	83.33	78.80	3.53	4.48
100	97.92	95.18	105.50	99.53	4.36	4.39
120	126.50	122.40	125.30	124.73	1.72	1.38

**Table 7:** Repeatability (system precision).

Ointment Concentration (% *w*/*w*)	Volume Applied (µL)	Theoretical Amount (ng/band)	Measured Amount (ng/band)	Nitrofurazone Yield (%)
0.20	4.00	80.00	80.93	101.16
0.20	4.00	80.00	83.01	103.76
0.20	4.00	80.00	80.34	100.43
0.20	4.00	80.00	84.11	105.14
0.20	4.00	80.00	81.96	102.45
0.20	4.00	80.00	84.43	105.54
	Average		82.46	103.08
	SD		1.67	
	%RSD		2.02	

**Table 8:** Robustness (changes in mobile phase composition).

Mobile Phase Composition	Theoretical Amount (ng/band)	% Recovery	R_F_ (Mean ± SD)
Toluene–acetonitrile–ethyl acetate–glacial acetic acid (6:1.6:2:0.1, *v*/*v*)	80	104.08	0.18 ± 0.03
	100	101.80	
	120	95.75	
Toluene–acetonitrile–ethyl acetate–glacial acetic acid (6:2.4:2:0.1, *v*/*v*)	80	103.68	0.18 ± 0.03
	100	103.30	
	120	99.92	

**Table 9:** Forced degradation studies.

Degradation Type	R_F_ Nitrofurazone	R_F_ Degradant	% Degradation
Photolysis and oxidation	0.188	-	16.24
Oxidation	0.188	0.735	14.21
Acid hydrolysis (0.1 M HCl)	0.185	0.008	19.29
Acid hydrolysis (0.5 M HCl)	0.185	0.008	24.37
Acid hydrolysis 1 M HCl)	0.185	0.008	25.38
Alkaline hydrolysis (0.01 M NaOH)	-	-	100
Alkaline hydrolysis (0.05 M NaOH)	-	-	100
Alkaline hydrolysis (0.1 M NaOH)	-	-	100

**Table 10:** Nitrofurazone content in different ointment preparation.

Theoretical Amount of Nitrofurazone in Ointment (ng/band)	Recovered Amount (ng/band)			Mean	SD	Nitrofurazone Yield (%)	Calculated Concentration of Nitrofurazone (% *w*/*w*)
	Run 1	Run 2	Run 3				
80	79.19	77.45	82.12	79.59	1.93	99.48	0.20
100	102.80	96.42	103.30	100.84	3.13	100.84	0.20
120	117.90	117.70	116.80	117.47	0.48	97.89	0.20

## Data Availability

The original contributions included in the study are presented within the article. Further inquiries can be sent to the corresponding author.

## Supplementary Materials

The following supporting information can be downloaded at: https://www.mdpi.com/article/10.3390/molecules30163429/s1, Figure S1: Images of TLC plates taken at 366 nm in three mobile phases: (a) formic acid–ethyl acetate–toluene (1:3:1); (b) formic acid–ethyl acetate–toluene (1:4:1); and (c) toluene–acetonitrile–ethyl acetate–glacial acetic acid (6:2:2:0.1). Spot NS: Nitrofurazone solution; Spot NO: Nitrofurazone ointment; and Spot SO: Blank simple ointment. R_F_s are indicated by the arrow.
