# Supplementary material for: Development and Validation of a Stability-Indicating HPTLC-Based Assay for the Quantification of Nitrofurazone Ointment
Source: Molecules. 2025 Aug 20;30(16):3429. doi: 10.3390/molecules30163429 (PMC12388184; doi:10.3390/molecules30163429)
Supplement: Supplementary file 1 [file molecules-30-03429-s001.zip › molecules-3731400-supplementary.pdf]

Supplementary Materials

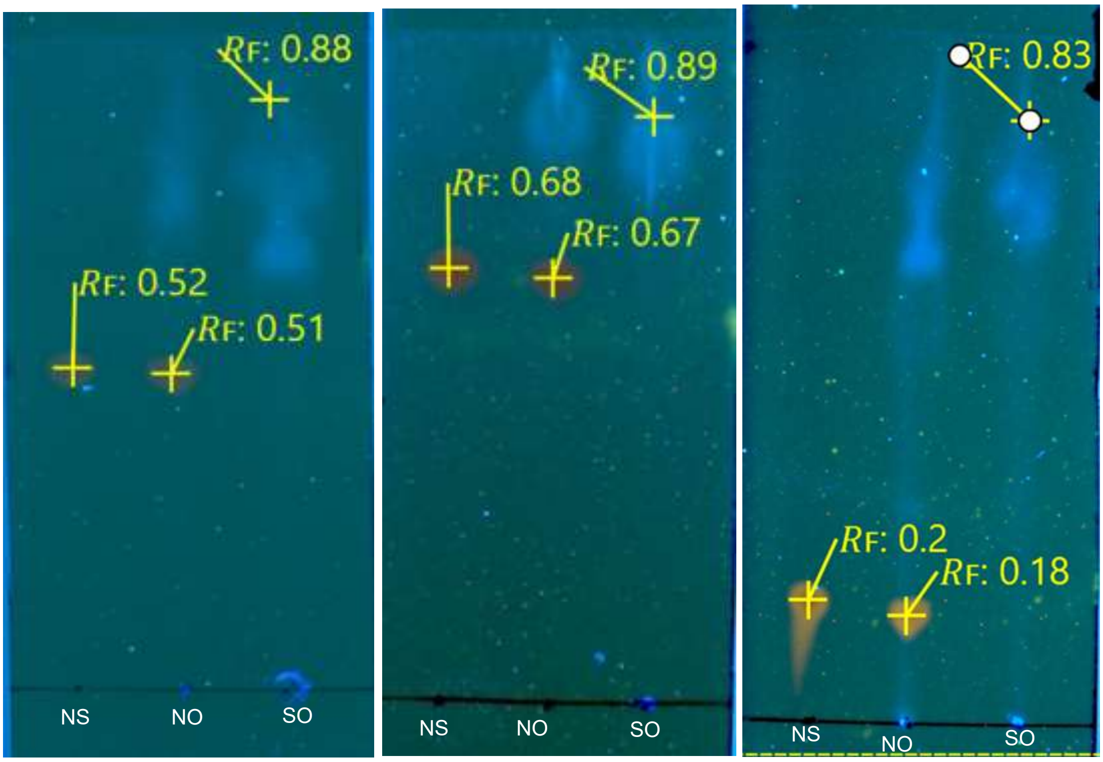

Figure S1: Images of TLC plates (normal silica gel plate) taken at 366 nm in three mobile phases: (a) Formic acid-ethyl acetate-toluene (1:3:1); (b) Formic acid-ethyl acetate-toluene (1:4:1) and (c) Toluene-acetonitrile-ethyl acetate-glacial acetic acid (6:2:2:0.1). Spot NS: nitrofurazone solution (30 μg/mL); Spot NO: nitrofurazone ointment (20 μg/mL); Spot SO: blank simple ointment. R_F's are indicated by the arrow.
